# From DGCR8 expression analysis to diseased pathways in 22q11.2 deletion syndrome

**DOI:** 10.3389/fimmu.2025.1611527

**Published:** 2025-11-24

**Authors:** Valentina Boz, Marianna Di Rosa, Alessia Pin, Eleonora De Martino, Flavio Faletra, Francesco Baldo, Andrea Magnolato, Erica Valencic

**Affiliations:** 1 Department of Paediatrics, Institute for Maternal and Child Health IRCCS “Burlo Garofolo”, Trieste, Italy; 2 Department of Medical, Surgical and Health Sciences University of Trieste, Trieste, Italy; 3 Institute of Medical Genetics, Azienda Sanitaria Universitaria Friuli Centrale, Udine, Italy; 4 Department of Medicine (DMED), University of Udine, Udine, Italy

**Keywords:** 22q11.2 deletion syndrome, *DGCR8*, miRNAs, immunophenotype, genotype, functional assays

## Abstract

22q11.2 deletion syndrome (22q11.2DS) is the most prevalent microdeletion disorder, distinguished by markedly diverse clinical manifestations, with its underlying molecular mechanisms not yet fully elucidated. This study sought to examine the expression of the commonly deleted gene *DGCR8* and its possible association with immune cell populations and cellular pathways involved in the immune response. We quantified *DGCR8* mRNA levels, evaluated immune cell subsets, and conducted functional assays to measure S6-phosphorylation (PI3K-AKT-mTOR activity), H2AX-phosphorylation kinetics (DNA damage response), and STAT1 expression (interferon response) in 13 pediatric patients compared with healthy controls. Our findings indicate a reduced expression of *DGCR8* in patients relative to controls, albeit with significant variability and notably low levels, as well as in one patient whose deletion did not include the *DGCR8* gene. A notable positive association between *DGCR8* expression and natural killer (NK) cell numbers was identified exclusively in the patient cohort, suggesting that NK cells may serve as a clinically biomarker for assessing microRNA dysregulation severity in 22q11.2DS. Nonetheless, the functional studies performed did not demonstrate substantial differences between patients and controls. In summary, this study validates altered *DGCR8* expression and establishes a novel association with NK cells in patients with 22q11.2DS. It implies that factors beyond mere gene deletion may affect *DGCR8* levels and highlights a positive correlation between *DGCR8* expression and NK cell count. The identification of this relationship has immediate clinical implications, as routine flow cytometric assays of NK cells could potentially provide a biomarker for monitoring immune dysfunction in patients with 22q11.2DS.

## 1 Introduction

The 22q11.2 deletion syndrome (22q11.2DS) is the most common microdeletion syndrome in humans, with an estimated incidence of 1:4000 live births. The phenotypic spectrum is highly variable, and over 190 different clinical manifestations have been reported, including congenital heart defects, palate abnormalities, immune system disorders, and hypoparathyroidism (1–4). A high degree of phenotypic variability has also been reported in patients from the same family (4). The molecular mechanisms underlying 22q11.2DS have not been fully clarified. The deletion varies in size from patient to patient and can lead to the loss of several genes. Among these, one of the most commonly deleted genes in patients is the DiGeorge Critical Region Gene 8 (*DGCR8*), which encodes an essential component of the microprocessor complex that contributes to the biogenesis of microRNAs (miRNAs, miR). Due to their regulatory action, miRNA imbalance can affect many cellular functions related to disease pathogenesis, especially concerning the development of the thymus (5, 6). A recent study revealed an altered expression of miR-185-5p in individuals with 22q11.2 deletion and *DGCR8* haploinsufficiency (7).

MiRNAs are known to be important for the immune system, influencing pathways like the PI3K-AKT-mTOR and interferon responses. It was recently shown that a complex regulatory network of microRNAs targets various components of the IFN pathway at multiple levels, suggesting that microRNAs act as important “buffers” to fine-tune immune responses with potential therapeutic and diagnostic applications (8). Based on the crucial role of miRNAs in B cell development, researchers investigated the PI3K-AKT-FOXO1 pathway. They found that a lack of miRNAs halts B cell development by increasing cell death and inhibiting proliferation. The study revealed that miRNAs are essential regulators of this pathway, which is vital for B cell maturation and antibody production (9).

Therefore, a lack of miRNAs might lead to the common immune problems in DiGeorge syndrome, such as an increased risk of viral infections, autoimmunity, and poor T and B cell immunity.

Additionally, Calses and colleagues have demonstrated the involvement of *DGCR8* in DNA repair following UV-induced damage (10). This aspect might help explain the increased incidence of cancer described by some authors in patients with del22q11.2 (11). This function in DNA repair may elucidate results from a study of 32 individuals with hereditary 22q11.2DS, which indicated an increased number of clinical symptoms and a more severe phenotype in the second generation (4). This worsening of the clinical phenotype could likely be due to the accumulation of additional genetic changes across generations, possibly favored by defective DNA repair.

Despite being known for over 50 years, the pathogenic mechanisms behind the clinical manifestations of this syndrome remain unclear. In this regard, investigating the relationship between deletion size and its associated features and the functional assessment of specific pathways could provide valuable insights for identifying new biomarkers and potential therapeutic targets. Our aim was to investigate less-explored biological activities in 22q11.2DS, specifically PI3K-AKT-mTOR activation, DNA repair, and type I interferon-related inflammation. We also sought to assess their connection to *DGCR8* expression levels.

## 2 Materials and methods

### 2.1 Patients and healthy donors

Patients, up to the age of 18, were enrolled after signing the informed consent, while healthy controls were adult volunteers and healthy pediatric subjects undergoing elective blood examinations.

The first group of healthy individuals (control group 1) was recruited concurrently with patients for functional assays. A second group of healthy volunteers (control group 2) was collected later to assess the correlation between *DGCR8* gene expression and lymphocyte subsets.

All patients had already received a molecular diagnosis of 22q11.2DS. We noted clinical phenotype and lymphocyte population values from the complete blood count (CBC) with differential of every patient.

For each patient and healthy control, we collected heparinized peripheral whole blood, part of which was used to obtain Peripheral Blood Mononuclear Cells (PBMCs), by stratifying through centrifugation on density gradient Ficoll (1,077 g/mL, Lympholyte, Cederlane). For prepubertal patients, we prioritized age-matched controls using blood specimens from healthy pediatric subjects undergoing elective blood examinations. When these age-matched pediatric samples weren’t available, or for adolescent patients, we obtained blood samples from adult volunteers.

### 2.2 Molecular diagnosis and deletion analysis

Molecular diagnosis was performed by SNP-array with the kit “HumanOmniExpress Exome BeadChip” Illumina (assembly hg19/GRCh37) in 10 patients and by CGH-array in 3 patients.

The DECIPHER database (https://www.deciphergenomics.org/) was consulted to analyze genes affected by the deletion in each patient and rank them estimated intolerance to haploinsufficiency, as expressed by the pLI metric.

### 2.3 Expression levels of *DGCR8*

Total RNA extraction was performed from 5x10^5 PBMCs using the Single Cell RNA Purification kit (Norgen Biotek Corp; Thorold, ON, Canada; Cat. N° 518000), according to the manufacturer’s instructions. The total RNA was eluted in 20 μL of elution buffer and stored at -80°C. The RNA quantity and purity were determined using a NanoDrop™ ND-1000 spectrophotometer (Thermo Fisher Scientific, Waltham, MA, USA).

A minimum of 200 ng to a maximum of 1000 ng of RNA was reverse transcribed into cDNA using the SensiFAST™ cDNA Synthesis Kit (Meiridian Bioscience^®^, Cincinnati, Ohio, USA; Cat. N° BIO-65054) following the manufacturer’s instructions.

As regards quantitative Real-time PCR, 25 ng of cDNA was added to 5 µl of 2X TaqMan™ Fast Advanced Master Mix (Thermo Fisher Scientific, Waltham, Massachusetts, USA; Cat. N°4444557), 0.5 µl of 20X probe (*DGCR8* Hs00987085_m1 or *G6PD* Hs00959073_g1 or *HPRT1* Hs02800695_m1; TaqMan^®^ Gene Expression Assay, Thermo Fisher Scientific, Waltham, Massachusetts, USA) and made up to a final volume of 10 µl with sterile water. Each reaction was carried out duplicate in a CFX96 Opus PCR Detection system (Bio-Rad, Hercules, CA, USA) using the following cycling condition: 2 min at 50°C, 10 min at 95°C, 40 cycles at 95°C for 15 s and 60°C for 1 min.

For relative quantification, the “delta-delta Ct” method (12) was applied, and *G6PD* and *HPRT1* were used as housekeeping genes. Furthermore, the expression of the *DGCR8* gene was assessed in separate control group 2.

### 2.4 Immunophenotype

Heparinized peripheral whole blood was collected from each patient and control participant in group 2 to assess immune cell phenotypes, including T lymphocyte subpopulations, B lymphocyte maturation profile, and CD169 (Siglec-1) expression on monocytes via flow cytometry (13). Surface markers analyzed are reported in **Supplementary Table S1** in the **Supplementary Materials** section. Recent Thymic Emigrants (RTE) are defined as CD3+CD4+CD31+CD45RA+. Recent Bone Marrow emigrants (RBE) were defined as CD19+ CD10+CD21-; B naïve are defined as CD19+CD27-IgD/M+; memory-IgM B cells were defined as CD19+CD27+IgD/M+; switched memory B cells were defined as CD19+CD27+IgD/M-. NK cells were defined as CD3-CD16/CD56+ cells.

### 2.5 S6 phosphorylation level

Freshly isolated PBMCs of patients and healthy controls, obtained as described above, were subject to various stimulations. To assess the phosphorylation level of the ribosomal protein S6 (a marker of PI3K/Akt/mTOR pathway activation), PBMCs were stimulated with PMA-ionomycin (Cell Stimulation Cocktail 500X, eBioscience) or Dynabeads Human T-activator CD3/CD28 (Gibco by Life Technologies) for 3 hours at 37°C, 5% CO2. Following stimulation, cells were surface stained with CD3 VioBlue (Miltenyi) and then fixed and permeabilized with paraformaldehyde 4% and methanol 90% respectively, before staining with anti-pS6 PE (Cell Signaling) and its respective isotype control.

### 2.6 H2AX phosphorylation level

To evaluate the cellular response and the kinetics of repair following induced DNA damage, thawed cells were stimulated with Bleomycin (9 µM, Sigma) for 1 hour at 37°C and 5% CO2, to induce DNA double-strand breaks. H2AX phosphorylation (γH2AX) was assessed at multiple time points: immediately following the 1-hour Bleomycin stimulation (end of stimulation) and after 3 and 20 hours from the washout of Bleomycin. While the direct “extent of DNA damage” is challenging to quantify precisely with γH2AX alone, the dynamic changes in γH2AX levels provide critical insights into the DNA damage response and subsequent repair processes. The initial phosphorylation (increase) of H2AX at serine 139 to form γH2AX, occurring rapidly at sites of DNA double-strand breaks (DSBs), serves as a sensitive marker for the induction of DNA damage. Conversely, the subsequent dephosphorylation (decrease) of γH2AX at later time points reflects the resolution of DNA damage and the progression of DNA repair pathways. The rate and efficiency of this dephosphorylation are indicative of the cell’s capacity to repair the induced lesions. Therefore, by analyzing the temporal profile of γH2AX phosphorylation and its subsequent decline, we evaluated the dynamic processes of DNA damage recognition, signaling, and repair following the experimental induction of DNA damage. Following stimulation, cells were surface stained with CD3 VioBlue (Miltenyi) and then fixed and permeabilized with paraformaldehyde 4% and methanol 90% respectively, before staining with anti-pH2AX Alexa Fluor 488 (BD Pharmingen) and its respective isotype control.

### 2.7 STAT1 expression

Thawed PBMCs of patients and healthy controls, obtained as described above, were treated with IFN2alpha (300 U/mL, Miltenyi Biotech) for 48 and 72 hours at 37°C, 5% CO2.

Cells were surface stained with CD3 VioBlue (Miltenyi), CD4 PE (Miltenyi) and CD45 V500-C (BD). Cells were then fixed and permeabilized with Fix&Perm Biolegend kit and stained with anti-STAT1 AF647 (BD) or corresponding isotype control AF647 (Biolegend).

### 2.8 Statistical analyses

Pearson correlations between various lymphocyte subpopulations (percentage and cell counts/µl) and *DGCR8* expression levels were performed in patients and control group 2 using the cor.test function from the stats package in the R environment. Correlation values ≥ 0.5 or ≥ - 0.5 and p-value < 0.1 will be considered. The use of a significance level of p<0.1 in our study is justified by the nature of the research, specifically our focus on a rare disease. In studies with small sample sizes, like those involving rare conditions, this can be justified potential to detect associations that might be missed with a stricter threshold like p<0.05.

Mann-Whitney test was performed using GraphPad Prism version 10.2.0.

## 3 Results

### 3.1 Patients enrolled

Patients: 13 (5 female, 8 male), with a median age of 7 years (average 7 years, range 8 months - 17 years).

Control Group 1: 17 subjects (9 female, 8 male), with a median age of 31 years (average 34 years, range 8–56 years). Their samples were collected at the same time as the patients’ for functional assays.

Control Group 2: 11 subjects (8 female, 3 male), with a median age of 31 years (average 32 years, range 2–45 years). Their samples were collected later to assess the correlation between *DGCR8* gene expression and lymphocyte subsets. Clinical and demographic data of all patients are summarized in **Table 1**.

**Table 1:** Clinical and demographic data of enrolled patients.

Patient	Sex	Age (years)	Congenital heart defects (CHD)	Autoimmune disorders	Allergy	Recurrent infections (other than otitis media)	Thymectomy	CD3 lymphopenia	Lymphatic proliferation
Pt1	F	13	no	no	no	no	no	no	no
Pt2	F	12	Bicuspid aorta	Thyroiditis	yes	yes	no	no	no
Pt3	F	6	Tetralogy of Fallot	Thyroiditis	no	no	yes	no	no
Pt4	M	3	Dilation of the aortic root	no	no	no	no	no	no
Pt5	M	1	Atrial Septal Defect	no	no	no	no	no	no
Pt6	M	17	no	Thyroiditis	no	no	no	no	no
Pt7	M	9	Tetralogy of Fallot	no	yes	no	yes	yes	no
Pt8	F	0.8	no	no	no	no	no	yes	no
Pt9	M	10	no	no	no	no	no	yes	Adenoidal hypertrophy
Pt10	M	0	no	no	no	no	no	no	no
Pt11	M	8	Right-sided aortic arch	no	no	no	no	no	Adenoidal hypertrophy
Pt12	M	9	Patent foramen ovale with a mild left-to-right shunt	no	no	no	no	no	no
Pt13	F	6	Atrial septal defect	no	no	no	no	no	no

Pt, patient; CHD, Congenital Heart Defects.

### 3.2 Deletion size and involved genes in patients

The deletion size among the included individuals varied from 1.0 to 3.0 megabases. In one individual, the deletions spared both *TBX1* and *DGCR8*, whose haploinsufficiency has been linked, respectively, with cardiac and neurological disease manifestations. **Table 2** reports a detailed analysis of the genetic characteristics of the included cases.

**Table 2:** Deletion size and probability of exhibiting haploinsufficiency in involved genes (according to the Decipher database).

Patient	Deletion detail	Deletion size	Deleted genes (probability of exhibiting haploinsufficiency ≥ 0.8)
Pt1	grch37:22:18874332-21490543	2.6 Mb	*HIRA*, *SCARF2*, *TBX1*, *DGCR8*, *MED15*
Pt2	grch37:22:18886915-21463730	2.6 Mb	*HIRA*, *SCARF2*, *TBX1*, *DGCR8*, *MED15*
Pt3	grch37:22:18661699-21661435	3.0 Mb	*HIRA*, *SCARF2*, *TBX1*, *DGCR8*, *MED15*
Pt4	grch37:22:18844632-21463730	2.6 Mb	*HIRA*, *SCARF2*, *TBX1*, *DGCR8*, *MED15*
Pt5	grch37:22:18900678-21304143	2.6 Mb	*HIRA*, *SCARF2*, *TBX1*, *DGCR8*, *MED15*
Pt6	grch37:22:18844632-21761763	2.9 Mb	*HIRA*, *SCARF2*, *TBX1*, *DGCR8*, *MED15*
Pt7	grch37:22:18886915-21463730	2.6 Mb	*HIRA*, *SCARF2*, *TBX1*, *DGCR8*, *MED15*
Pt8	grch37:22:19036998-21440514	2.4 Mb	*HIRA*, *SCARF2*, *TBX1*, *DGCR8*, *MED15*
Pt9	grch37:22:18747717-21630770	2.9 Mb	*HIRA*, *SCARF2*, *TBX1*, *DGCR8*, *MED15*
Pt10	grch37:22:20597993-21630770	1.0 Mb	*SCARF2*, *MED15*
Pt11	grch37:22:18912678-21463730	2.6 Mb	*HIRA*, *SCARF2*, *TBX1*, *DGCR8*, *MED15*
Pt12	grch37:22:18888203-21463041	2.6 Mb	*HIRA*, *SCARF2*, *TBX1*, *DGCR8*, *MED15*
Pt13	grch37:22:18919528-21460595	2.5 Mb	*HIRA*, *SCARF2*, *TBX1*, *DGCR8*, *MED15*

Pt, patient.

### 3.3 Expression levels of *DGCR8* and correlation with immunophenotype

The expression of *DGCR8* was assessed by comparing the fold changes in patients to those observed in healthy donors. Results indicated that *DGCR8* expression was higher in the control group (p<0.0001).

The Pearson test was conducted to connect the expression levels of the DGCR8 gene with various lymphocyte subpopulations (CD3, RTE, CD4, CD8, CD19, and CD16/CD56, calculated as percentages and absolute count) in both patients and controls (control group 2).

**Figure 1:**
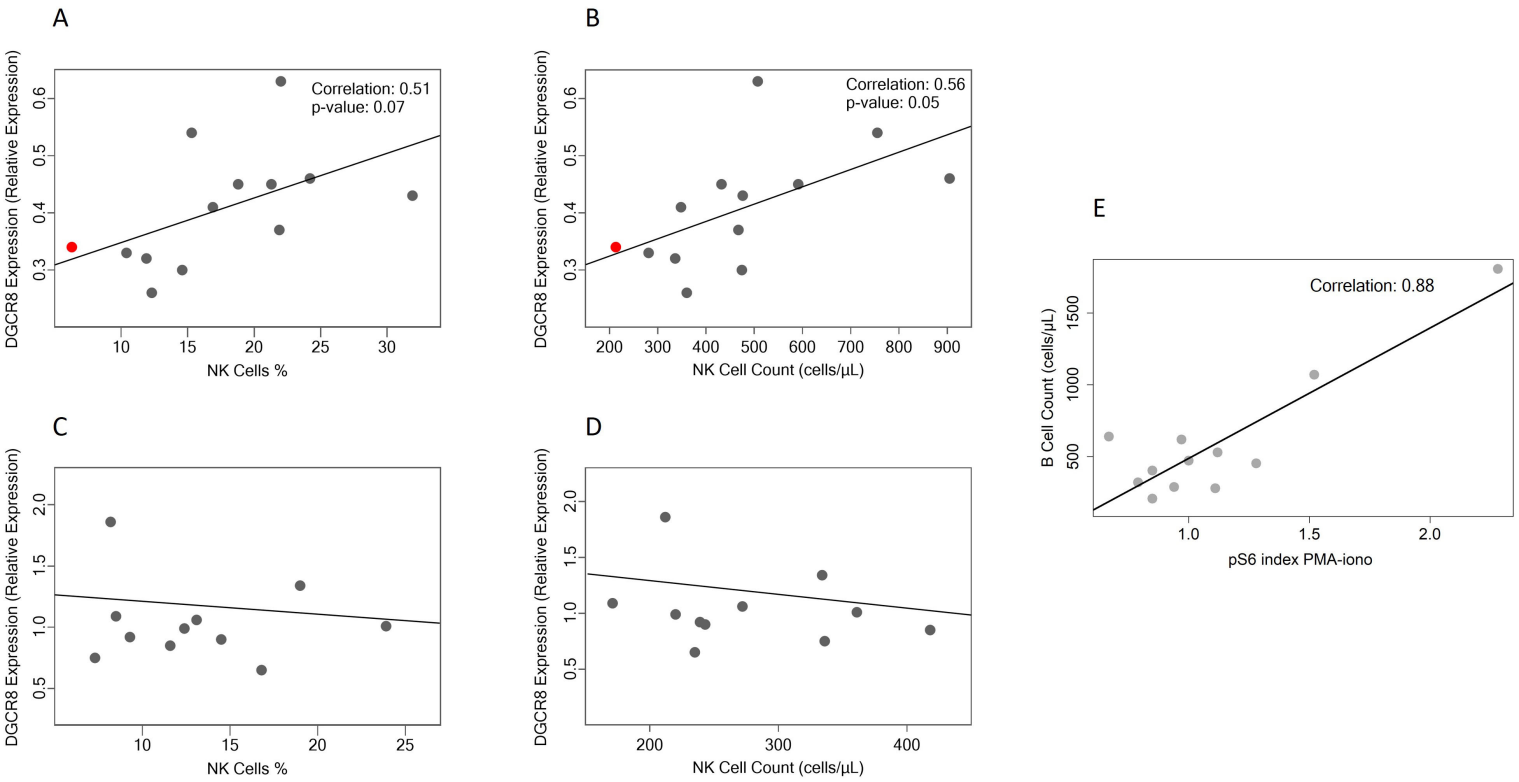
**(A–D)** Scatter plot showing the correlation between *DGCR8* gene expression levels and NK cell, as percentage (A, patients; C, controls), and as cell count (B, patients; D, controls). The red dots indicate the patient without *DGCR8* deletion. **(E)** Correlation between the activation of PI3K pathway assessed by measure of S6 phosphorylation, after PBMCs stimulation and absolute counts of B cells. S6 phosphorylation was not performed in patient without *DGCR8* deletion. Abbreviations: pS6 (phosphorylated S6 protein); PMA-iono (phorbol-12-myristate-13-acetate and ionomycin).

Overall, correlation values of ≥ 0.5 or ≥ -0.5 were noted for CD3 (cor = -0.50, p = 0.09), RTE (cor = -0.50, p = 0.08), CD4 (cor = -0.50, p = 0.07), and CD16/CD56 (cor = 0.51, p = 0.07; **Figure 1A**) when considering cell populations as a percentage in the patient group. A significant direct association was observed with natural killer cell count, resulting in a correlation coefficient of 0.56 and a p-value of 0.05 (**Figure 1B**). The red dot indicates the patient without *DGCR8* deletion. No significant correlations were observed in control group 2 between the *DGCR8* gene and lymphocyte subpopulations (**Figures 1C, D**).

### 3.4 Immunophenotype

The analysis of lymphocyte subpopulations did not reveal any major defects, except for the evident increase in the percentage of NK cell population in patients 7 and 8 and for the significant decrease of total B cells, both as percentage and absolute number, in patient 10 (**Supplementary Tables S2**, **S3**). Recent thymic emigrants were within normal range, also in the two patients who underwent major cardiac surgery with partial or total thymectomy. The expression of Siglec-1 on monocytes was increased in 5 patients, three of whom exhibited symptoms consistent with a viral infection at the time of the sample collection.

### 3.5 S6 phosphorylation level

**Figure 2:**
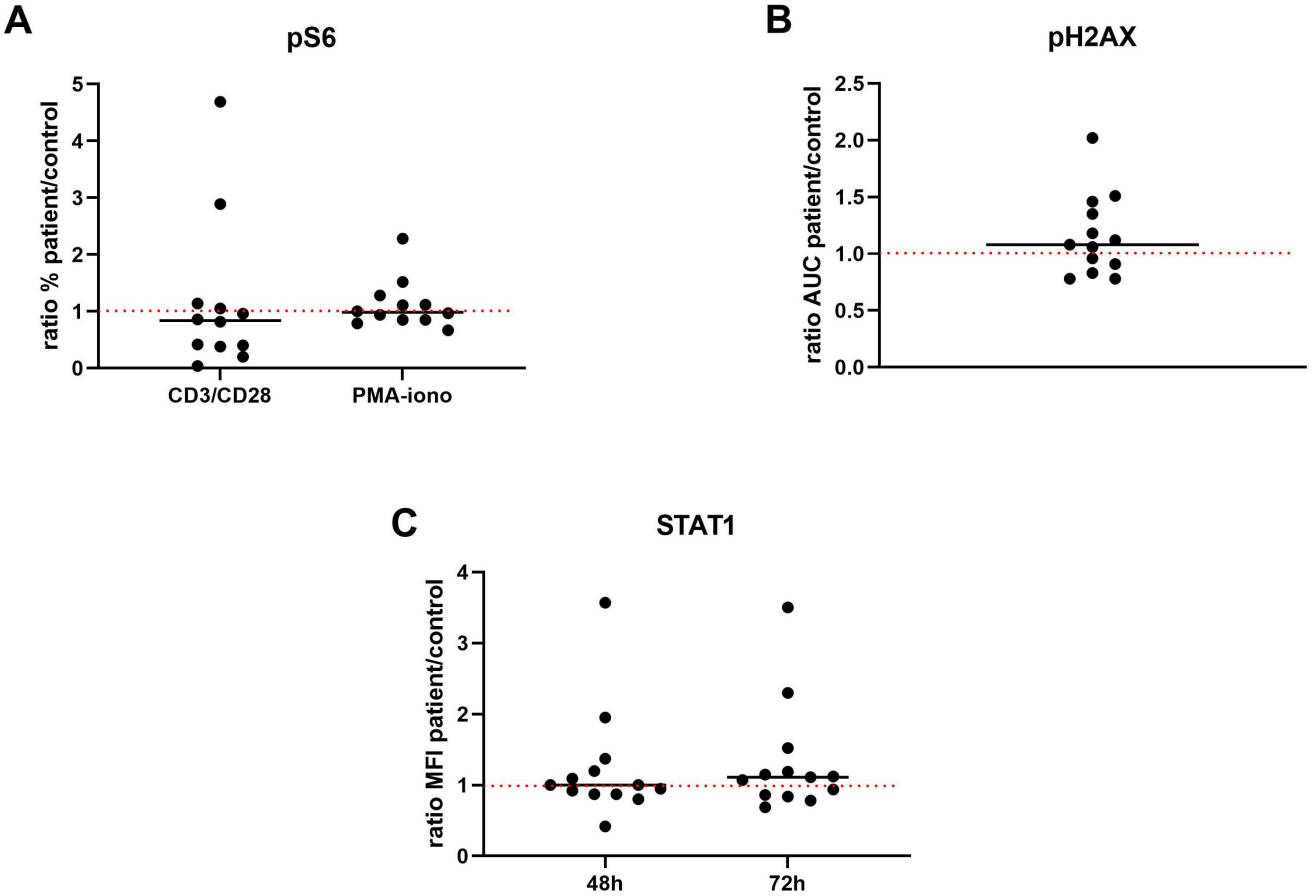
**(A)** Scatter plot showing the percentage of cells phosphorylating S6 for two stimulation conditions: PMA-ionomycin and Dynabeads Human T-activator CD3/CD28. For each experimental condition, data are presented as the ratio of the percentage observed in the patient to that observed in the corresponding healthy donor. **(B)** Scatter plot illustrating the kinetics of γH2AX phosphorylation in patients versus controls, reflecting DNA damage response by assessing both the extent of damage and the efficiency of repair. γH2AX phosphorylation levels were measured at several time points post-bleomycin treatment: immediately after the 1-hour stimulation, and at 3- and 20-hours following drug removal. These data were used to calculate the Area Under the Curve (AUC), providing a comprehensive summary of the response dynamics. The AUC ratio (patient AUC divided by control AUC) is displayed for each case. **(C)** Scatter plot showing how a patient’s immune cells respond to stimulation compared to those of a healthy subject. Specifically, each point represents the ratio of phosphorylated STAT1 levels (measured by mean fluorescence intensity, MFI) in the patient’s cells to the corresponding levels in healthy control cells. The dashed line at a ratio of 1 indicates similar responses between patients and controls. pS6, phosphorylated S6 protein; PMA-iono, phorbol-12-myristate-13-acetate and ionomycin; AUC, Area Under the Curve; MFI, mean fluorescence intensity.

To assess a potential effect of altered expression of certain miRNAs on the activation of the PI3K-AKT-mTOR pathway, we measured S6 phosphorylation in resting and stimulated PBMC using flow cytometry. The data, quantified as the percentage of cells phosphorylating S6, were initially normalized to the isotype control and the resting condition, subsequently represented as the ratio between the values obtained from the patient and the corresponding healthy donor in each experimental condition (pS6 index). Overall, S6 phosphorylation in patient lymphocytes did not differ from controls (**Figure 2A**). However, within the patient cohort, those with higher activation of PI3K pathway after PBMCs stimulation, as assessed by measure of S6 phosphorylation, tended to have higher percentages and absolute counts of B cells (**Figure 1E**).

### 3.6 H2AX phosphorylation

Given that a higher cancer risk has been reported associated with 22q11.2DS, we examined DNA damage response in our patient sample. To assess both the cellular response to DNA damage induction and the subsequent repair efficiency, we evaluated the kinetics of γH2AX phosphorylation following the treatment of patient peripheral blood cells with bleomycin. Specifically, γH2AX phosphorylation levels were measured at multiple, distinct time intervals after the addition of bleomycin: immediately following the 1-hour stimulation, and then 3 and 20 hours after bleomycin washout. These time-dependent measurements provided a comprehensive profile of the DNA damage response, capturing the initial phosphorylation event and the subsequent dephosphorylation reflecting repair. The overall dynamic process was quantitatively summarized by the area under the curve (AUC) derived from these time-series γH2AX phosphorylation data. To compare the DNA damage response between patients and controls, we represented the data as the patient AUC/control AUC ratio (referred to as the pH2AX index). We demonstrated that the AUC for participants with 22q11.2DS did not substantially vary from that of the controls, as evidenced by a pH2AX index close to 1 (**Figure 2B**, **Supplementary Figure S1**).

### 3.7 STAT1 expression

The potential imbalance in the interferon response was assessed through the expression of Siglec-1 on monocytes in peripheral whole blood and in *ex vivo* tests by assessing STAT1 activation in IFN2α stimulated PBMCs. We normalized the data, expressed as the median fluorescence intensity (MFI) of STAT1, to both the isotype control and the resting condition. We thus represented results as the ratio between the value obtained in the patient and that obtained in the paired healthy donor in each experimental condition (STAT1 index). We showed that there are no substantial differences between patients and healthy controls as evidenced by a STAT1 index close to 1 both after 48h and 72 h of stimulation (**Figure 2C**).

## 4 Discussion

22q11.2 deletion syndrome is a complex genetic condition characterized by significant clinical variability, making its molecular basis challenging to understand. While congenital heart defects and immune system problems are hallmarks, the syndrome encompasses a wide range of symptoms, including neurodevelopmental issues and increased cancer susceptibility. This diversity points to the intricate roles of genes within the deleted chromosomal region. Among these, *DGCR8* attracts considerable interest due to its function in processing primary miRNA transcripts, which in turn regulate numerous genes potentially influencing the disease’s presentation. This study aimed to explore several less-investigated biological activities and their possible connection to *DGCR8* expression levels in individuals with 22q11.2DS.

Our research involved clinical, immunophenotypic, and molecular analyses of peripheral blood samples collected from patients consecutively referred to our institution with a confirmed 22q11.2DS diagnosis.

First, we examined the *DGCR8* expression. Compared to healthy controls, patients with 22q11.2DS generally showed lower *DGCR8* levels. This aligns with previous research attributing reduced expression primarily to haploinsufficiency when the deletion directly includes the *DGCR8* gene itself (14). However, our data revealed particularly low expression even in one individual (patient #10) whose smaller deletion did not encompass the *DGCR8* gene. We can speculate that this finding suggests that reduced *DGCR8* expression in 22q11.2DS can occur independently of its physical deletion, pointing toward complex regulatory mechanisms contributing to the syndrome. A potential explanation can be the disruption of distant regulatory elements (enhancers, repressors) or transcription factors that control *DGCR8*. Indeed, deletion size is not considered the main driver of clinical variability (15). Furthermore, alterations in local chromatin structure or DNA methylation patterns due to the deletion near *DGCR8* could silence the gene despite its remaining intact (16). Additionally, feedback loops with miRNAs that are not working properly and are known to change in 22q11.2DS (like *miR-185-5p* and *miR-1304-3p*) could directly affect *DGCR8* mRNA.

Another noteworthy new exploratory finding was the positive correlation between *DGCR8* expression and peripheral NK cell counts, specifically in patients with 22q11.2DS. Although NK cell numbers are not directly tied to thymic hypoplasia, abnormalities in this immune cell subset have been reported in the syndrome. Therefore, while this remains a univariate correlation and we cannot definitively attribute it solely to DGCR8 deletion, the observed link between lower *DGCR8* expression and reduced NK cell counts warrants further investigation. Interestingly, despite the wide range of *DGCR8* levels in healthy controls, we found no such correlation between *DGCR8* expression and NK cells in this group. The identification of this NK cell-*DGCR8* relationship has immediate clinical implications, as routine flow cytometric analysis of NK cell populations could potentially provide a practical biomarker for monitoring immune dysfunction in pediatric patients with 22q11.2 deletion syndrome. Multivariate analysis on larger series can help disentangle the contribution of other genes within the deletion.

Importantly, reduced DGCR8 expression was also occasionally observed in healthy controls, indicating a role for trans-acting regulatory elements, epigenetic modifications, or feedback from miRNAs themselves. This suggests that, while haploinsufficiency may increase risk, DGCR8 reduction alone is insufficient to produce the full phenotype, which instead likely results from multifactorial interactions between gene dosage and additional regulatory mechanisms.

We also analyzed other cellular functions potentially relevant to 22q11.2DS pathogenesis or comorbidities.

Individuals with 22q11.2DS often exhibit variable B lymphocyte deficiencies unrelated to thymic problems. Earlier studies that removed DGCR8 completely from B cells found that it increased PTEN activity, which reduced the activity of the PI3K-AKT-mTOR pathway and impaired B cell maturation (9). To assess if haploinsufficiency impacts this pathway in our patients, we measured S6 protein phosphorylation (a marker of PI3K activity) in peripheral T lymphocytes following receptor or polyclonal stimulation (17, 18). We found no significant difference in the response between patients and healthy controls. However, among patients, those with higher phosphorylation of S6 compared to their paired control tended to display higher percentages and counts of B cells. In general, our findings did not show a link between *DGCR8* expression and S6 phosphorylation levels after T cell stimulation, but they did not dismiss the idea that problems with PI3-AKT-mTOR could play a role in the immune imbalance related to the disease.

We also investigated the expression of Siglec-1 on peripheral monocytes as a marker of type I interferon-related inflammation (13). Even though the differences between controls and patients was not significant, five individuals with an enhanced Siglec-1 expression may be consistent with the inflammatory burden of common illnesses in 22q11.2DS (19), even if we cannot exclude a contribution of possible vascular disorders on this parameter (20).

Given the known increased cancer risk in 22q11.2DS (21), we examined the DNA damage response in our patient cohort. To assess how these patients respond to and repair DNA damage, we treated their peripheral blood cells with bleomycin and monitored the kinetics of γH2AX phosphorylation. This allowed us to evaluate both the initial cellular response to DNA damage induction and the efficiency of the subsequent repair process by observing the dynamic changes in γH2AX levels over time. The area under the curve (AUC) representing these kinetics did not differ significantly when comparing our patients and controls. This observation of a normal DNA damage response within the 22q11.2DS cohort is potentially relevant as it suggests that the increased cancer incidence reported in some studies of 22q11.2DS patients is unlikely to be attributable to defective DNA repair mechanisms. This finding is also potentially relevant when selecting medications for managing comorbidities, indicating that standard DNA-damaging agents are likely to be tolerated by these patients similarly to how they are by individuals without 22q11.2DS.

An established heightened risk of rheumatological disorders exists in 22q11.2DS (22). We investigated potential underlying interferon-associated inflammation. While some patients showed elevated Siglec-1 levels on monocytes (an indicator of interferon activity), this finding could have been confounded by recent viral infections in those individuals. Crucially, examining STAT1 phosphorylation after stimulation with interferon-alpha revealed a normal response compared to controls. These data suggest that pre-existing, systemic interferon-induced inflammation is unlikely to be the primary driver of the reported rheumatological risk in these patients. Clinically, this suggests that the rheumatological manifestations in 22q11.2DS likely stem from other immune dysregulation mechanisms, guiding clinicians toward alternative diagnostic and therapeutic strategies rather than interferon-based interventions. While, these results are largely negative, they represent the first investigation of these specific pathways in patients with 22q11.2DS, contributing to a more complete mechanistic understanding of the syndrome.

In summary, this study underscores the molecular complexity of 22q11.2DS. We observed generally lower *DGCR8* expression in patients, potentially regulated by mechanisms beyond direct gene deletion and found a correlation with NK cell counts specific to this group. However, significant expression variability exists, implying interactions with other factors. Notably, our investigations into PI3K-AKT-mTOR pathway activity, DNA damage response kinetics, and interferon pathway signaling did not reveal significant differences between patients and controls in this cohort. These findings suggest these specific pathways might not be the main drivers of the immune dysfunctions or predispositions to cancer and rheumatological disorders as measured here, particularly considering the characteristics of our study population.

Understanding the complex interplay of factors influencing *DGCR8* expression and its impact on NK cell populations provides avenues for future research into targeted interventions. While our study highlights the resilience of certain pathways in this cohort, the observed heterogeneity in *DGCR8* levels and its implications for immune function emphasize the need for continued investigation into patient-specific molecular profiles to optimize care.

A potential bias in our study is represented by the lower number of patients who underwent cardiac surgery (2/13 participants) (23). This may have resulted in fewer cases of severe immunodeficiency in our cohort, since this condition can worsen when thymectomy is performed during cardiac surgery (24, 25). Furthermore, in our analysis, recurrent otitis media episodes in infancy—unless persistent or associated with other infection types—were not considered markers of immunodeficiency, since they are common in children with normal immune function and largely attributable to anatomical features typical of 22q11.2DS.

Our study has a few limitations to acknowledge. First, the inherent variability in the specific genes affected within the 22q11.2 deleted region among patients could potentially limit the broad generalizability of our findings. However, it’s quite intriguing that many features of 22q11.2DS consistently appear, irrespective of the deletion’s length or the genes involved. This consistency highlights the likely significance of altered gene regulation as a common underlying mechanism in 22q11.2DS, suggesting a wider impact than simply the direct loss of individual genes.

Secondly, our study was conducted with a relatively small sample size and at a single center. Despite this, the sample was sufficient to perform descriptive analyses and provide foundational insights into the biological activities studied.

Finally, we faced the challenge of suboptimal age-matching for our controls. To mitigate this, we used a pragmatic strategy: we ensured stricter age-matching for prepubertal children, while for our adolescent cohort, we more frequently used adult volunteer controls. For most of the parameters we analyzed, this approach is acceptable, as these parameters typically don’t show significant variability over a few years of age. Nevertheless, we cannot entirely rule that some of our results may have been influenced by this less-than-ideal age-matching.

Future research should prioritize dissecting the complex mechanisms controlling *DGCR8* expression independent of its deletion and exploring epigenetic factors and miRNA interactions. Clarifying the functional link between *DGCR8* levels and NK cell counts in patients is also crucial.

## Data Availability

All data generated or analyzed during this study are available upon request to the corresponding author.
