# Supplementary material for: From DGCR8 expression analysis to diseased pathways in 22q11.2 deletion syndrome
Source: Front Immunol. 2025 Nov 24;16:1611527. doi: 10.3389/fimmu.2025.1611527 (PMC12682781; doi:10.3389/fimmu.2025.1611527)

Supplementary Material

Table S1. Features of antibodies used in flow-cytometric panels: antigen recognized, fluorochrome conjugated and corresponding firm.

| **Lymphocyte subpopulation** | **Antigen** | **Fluorochrome** | **Firm** |
| --- | --- | --- | --- |
| BASIC LYMPHOCYTE SUBPOPULATIONS | CD3 | VioBlue | Miltenyi |
|  | CD45 | V500-C | BD |
|  | CD16 | Viobright515 | Miltenyi |
|  | CD56 | Viobright515 | Miltenyi |
|  | CD31 | PE | Miltenyi |
|  | CD8 | PerCP-Cy5.5 | BD |
|  | CD45RA | PE-Vio-770 | Miltenyi |
|  | CD4 | APC | BD |
|  | CD19 | APC-Cy7 | BD |
| PHENOTYPE B CELLS | CD21 | VioBlue | Miltenyi |
|  | CD45 | V500-C | BD |
|  | CD38 | FITC | BD |
|  | IgD | PE | Miltenyi |
|  | IgM | PE | Miltenyi |
|  | IgG | PerCP-Vio770 | Miltenyi |
|  | CD10 | PE-Vio770 | Miltenyi |
|  | CD27 | APC | Miltenyi |
|  | CD19 | APC-Cy7 | BD |
| CD169  (Siglec-1) | CD14 | VioBlue | Miltenyi |
|  | CD169 (Siglec-1) | APC | Miltenyi |
|  | IgG1 isotype control | APC | Miltenyi |

Table S2. Data of basic immune cell phenotype and T lymphocyte subpopulations for each patient. The reference ranges (min-max) for each lymphocyte population are indicated in brackets and values outside this range are shown in bold. References are derived from van Gent 2009 and from internal healthy pediatric/adult control population. Recent Thymic Emigrants (RTE) are defined as CD3+CD4+CD31+CD45RA+.

| **Patient** | **Leucocytes (cell/ul)** | **CD45++**  **(cell/ul)** | **CD45++**  **(%)** | **CD3+  (% of CD45++)** | **CD3+  (cell/uL)** | **RTE  (% of CD4+)** | **RTE  (cell/uL)** | **CD4+**  **(% of CD45++)** | **CD4+ (cell/uL)** | **CD8+**  **(% of CD45++)** | **CD8+ (cell/uL)** | **CD16/CD56+**  **(% of CD45++)** | **CD16/CD56+ (cell/uL)** |
| --- | --- | --- | --- | --- | --- | --- | --- | --- | --- | --- | --- | --- | --- |
| Pt1 | 6060  (4500-13000) | 2300  (1500-5200) | 37.0  (24.2-51.3) | 61.7  (40.1-68.0) | 1418  (1000-2200) | 38.9  (33.5-58.2) | 340  (240-480) | 38  (30.4-52.9) | 873  (600-1200) | 18.9  (13.6-23.5) | 435  (230-580) | 18.8  (3.0-22.0) | 432  (70-480) |
| Pt2 | 8150  (4500-13000) | 2060  (1500-5200) | 25.3  (24.2-51.3) | 64.4  (40.1-68.0) | 1328  (1000-2200) | **25.8**  (33.5-58.2) | **199**  (240-480) | 37.4  (30.4-52.9) | 770  (600-1200) | 19.3  (13.6-23.5) | 397  (230-580) | 16.9  (3.0-22.0) | 348  (70-480) |
| Pt3 | 7850  (4500-13500) | 2780  (1500-6800) | 35.4  (26.0-49.6) | 55.6  (43.0-63.0) | 1546  (1200-2600) | **40.1**  (43.9-66.4) | 388  (250-900) | 34.8  (26.5-41.4) | 968  (640-1400) | 17.5  (13.8-28.8) | 487  (250-1200) | **21.3**  (4.0-17.0) | **591**  (100-480) |
| Pt4 | 7860  (4500-13500) | 2930  (2000-8000) | 37.3  (36.3-60.5) | 64.3  (46.2-67.8) | 1885  (1400-3700) | 60.7  (52.7-73.9) | 829  (300-1500) | **46.6**  (18.3-42.7) | 1365  (650-2300) | **14.1**  (14.7-27.2) | 414  (370-1300) | 12.3  (4.0-17.0) | 360  (130-720) |
| Pt5 | 8870  (6000-13000) | 4930  (3000-9500) | 55.6  (43.0-65.2) | **44.5**  (48.0-70.1) | 2194  (1900-5900) | 61.7  (45.5-70.7) | **789**  (790-1600) | **25.9**  (33.0-55.0) | **1279**  (1900-3000) | 15  (14.0-26.0) | 741  (670-1500) | **15.3**  (3.0-15.0) | 755  (180-920) |
| Pt6 | 6780  (4000-11000) | 2310  (1000-4800) | 34.1  (27.0-44.1) | 62.3  (41.5-68.0) | 1438  (1000-2200) | **30.4**  (32.7-60.9) | 239  (200-590) | 34  (29.3-52.9) | 785  (560-1100) | **24.3**  (13.5-22.0) | **560**  (200-500) | 22  (3.0-22.0) | **507**  (70-480) |
| Pt7 | 13450  (4500-13500) | **1490**  (1500-6500) | **11.1**  (34.5-48.2) | 49.5  (43.0-63.0) | **738**  (1200-2600) | 43.4  (40.1-54.8) | **216**  (250-790) | 33.4  (28.4-44.4) | **498**  (700-1500) | **13.2**  (16.4-36.2) | **197**  (250-1000) | **31.9**  (4.0-17.0) | 476  (100-480) |
| Pt8 | 6330  (6000-18000) | **3740**  (4000-10500) | 59.1  (48.4-76.5) | **44.5**  (48.0-75.0) | **1664**  (1900-5900) | **51.2**  (54.8-75.3) | **627**  (790-2600) | 32.7  (29.8-63.4) | **1223**  (1300-4000) | **8.1**  (11.0-17.8) | **302**  (590-1500) | **24.2**  (3.0-15.0) | 905  (160-950) |
| Pt9 | 6710  (4500-13500) | 2130  (1500-5200) | **31.7**  (34.5-48.2) | 55.1  (43.0-63.0) | **1173**  (1200-2600) | **37.4**  (40.1-54.8) | **238**  (250-790) | 29.9  (28.4-44.4) | **636**  (700-1500) | 17.2  (16.4-36.2) | 365  (250-1000) | **21.9**  (4.0-17.0) | 467  (100-480) |
| Pt10 | 10130  (7900-13400) | **3400**  (4000-10500) | **36.1**  (50.1-70.1) | **85.4**  (50.0-74.0) | 2905  (2500-6500) | 70.2  (56.3-76.4) | 1345  (780-2700) | **56.3**  (37.6-46.4) | 1915  (1300-4000) | **26.6**  (7.7-22.0) | 903  (390-1900) | 6.3  (3.0-18.0) | 213  (170-950) |
| Pt11 | 8310  (4500-13500) | 2830  (1500-6500) | 34.1  (26.0-49.6) | **68.4**  (43.0-63.0) | 1935  (1200-2600) | 54.2  (43.9-66.4) | 467  (250-900) | 30.5  (26.5-41.4) | 862  (640-1400) | 24.6  (13.8-28.8) | 696  (250-1200) | 11.9  (4.0-17.0) | 336  (100-480) |
| Pt12 | 9650  (4500-13500) | 2690  (1500-6500) | **27.9**  (34.5-48.2) | **75.7**  (43.0-63.0) | 2038  (1200-2600) | **57**  (40.1-54.8) | 646  (250-790) | 42.1  (28.4-44.4) | 1133  (700-1500) | 27.7  (16.4-36.2) | 746  (250-1000) | 10.4  (4.0-17.0) | 281  (100-480) |
| Pt13 | **16590**  (4500-13500) | 3250  (1500-6800) | **19.6**  (26.0-49.6) | 62.6  (43.0-63.0) | 2035  (1200-2600) | 60  (43.9-66.4) | 796  (250-900) | 40.8  (26.5-41.4) | 1325  (640-1400) | 18.4  (13.8-28.8) | 598  (250-1200) | 14.6  (4.0-17.0) | 474  (100-480) |

Table S3. Data of maturation profile of B lymphocytes and the expression of CD169 (Siglec-1) on monocytes for each patient. The reference ranges (min-max) for each lymphocyte population are indicated in brackets and values outside this range are shown in bold. References are derived from van Gent 2009 and from internal healthy pediatric/adult control population. Recent Bone Marrow emigrants (RBE) are defined as CD19+ CD10+CD21-; B naïve are defined as CD19+CD27-IgD/M+; memory-IgM B cells are defined as CD19+CD27+IgD/M+; switched memory B cells are defined as CD19+CD27+IgD/M-.

| **CD19+**  **(% of CD45++)** | **CD19+**  **(cell/uL)** | **RBE**  **(% of CD19+)** | **B naive**  **(% of CD19+)** | **memory IgM**  **(% of CD19+)** | **switched memory**  **(% of CD19+)** | **CD169**  **(Siglec-1.**  **% of CD14+)** |
| --- | --- | --- | --- | --- | --- | --- |
| 17.5  (7.8-23.7) | 402  (120-580) | **1.1**  (1.5-7.3) | **84**  (64.6-80.1) | 5.8  (4.7-22.2) | 6  (3.3-14.2) | **51.4**  (>17) |
| 15.5  (7.8-23.7) | 320  (120-580) | 2.3  (1.5-7.3) | 72.8  (64.6-80.1) | 13  (4.7-22.2) | 8.9  (3.3-14.2) | 4.5  (>17) |
| 19  (8.5-20.2) | 529  (280-790) | **11.2**  (3.4-9.0) | **72.5**  (47.8-69.8) | **5.6**  (6.3-22.0) | 6.5  (1.8-14.2) | **30.7**  (>17) |
| 21.1  (14.4-25.1) | 618  (360-1500) | 12.7  (6.4-13.9) | 71.8  (49.7-77.1) | 8.1  (4.8-16.1) | 5.1  (1.5-9.7) | **17.7**  (>17) |
| **36.7**  (16.3-26.8) | **1809**  (870-1700) | 23.3  (9.7-26.7) | 60.9  (57.5-84.7) | 8.6  (4.6-15.0) | 4.3  (0.7-5.9) | 13.7  (>17) |
| 12.1  (7.8-15.1) | 279  (120-440) | 3.1  (1.5-7.3) | 76.9  (59.0-81.1) | 9  (4.6-23.8) | 5.6  (3.0-16.3) | 10.8  (>17) |
| 13.9  (4.3-18.2) | 207  (120-550) | **1.4**  (2.9-9.0) | **75.1**  (51.5-73.3) | 12.4  (6.5-22.2) | 6.7  (2.8-14.9) | 15.9  (>17) |
| 28.6  (11.1-45.4) | 1071  (900-3700) | **34.6**  (9.3-26.7) | **57.2**  (57.5-84.7) | 4.8  (4.6-15.0) | 2.5  (0.7-5.9) | **93.1**  (>17) |
| **21.2**  (4.3-18.2) | 452  (120-550) | 6  (2.9-9.0) | 63.5  (51.5-73.3) | 14.9  (6.5-22.2) | 10.5  (2.8-14.9) | 6  (>17) |
| **4.3**  (20.5-40.9) | **147**  (970-3700) | ND | ND | ND | ND | **30.4**  (>17) |
| 16.7  (8.5-20.2) | 472  (280-790) | **2.8**  (3.4-9.0) | **88**  (47.8-69.8) | **4.9**  (6.3-22.0) | **2.2**  (1.8-14.2) | 10.3  (>17) |
| 10.7  (4.3-18.2) | 287  (120-550) | 5  (2.9-9.0) | 73.3  (51.5-73.3) | 12.4  (6.5-22.2) | 5.7  (2.8-14.9) | 8.5  (>17) |
| 19.7  (8.5-20.2) | 640  (280-790) | 3.7  (3.4-9.0) | **71.9**  (47.8-69.8) | 9.1  (6.3-22.0) | 8.8  (1.8-14.2) | **26.7**  (>17) |

Figure S1. Kinetics of γH2AX phosphorylation after bleomycin (bleo) treatment in single patients compared with control groups. Percentage of pH2AX positive cells at each time point. Legend. ns t0: untreated cells at baseline; bleo t0: cells after 1 hour of bleomycin incubation; bleo t1: cells after 3 hours of bleomycin withdrawal; bleo t2: cells after 20 hours of bleomycin withdrawal. Green line: average of controls (with SD); red line: patient (Pt).


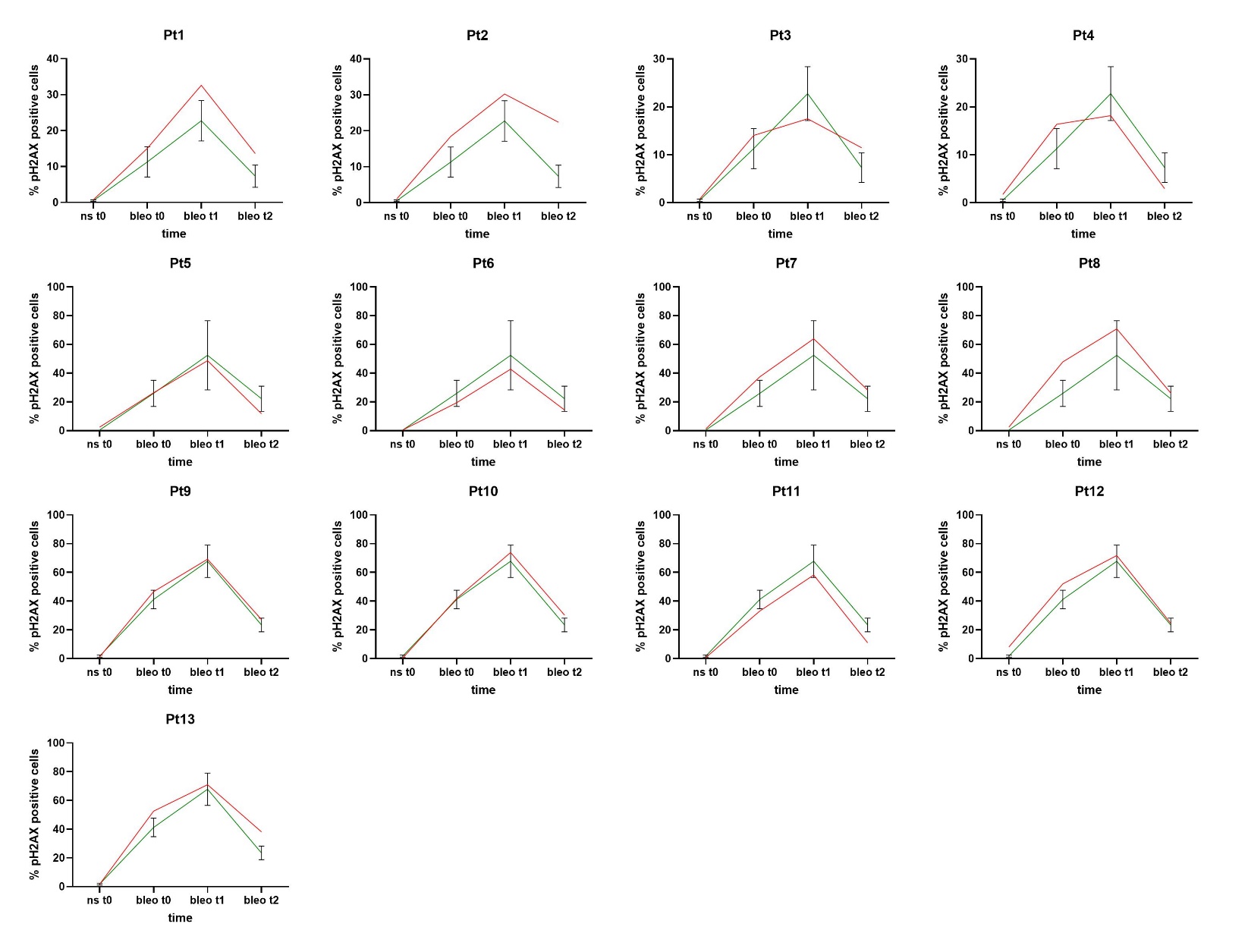

Supplement: Supplementary file 1 [file Table1.docx]
